# Validation of large animal models in mechanical valve research: a histologic comparison

**DOI:** 10.1093/icvts/ivae070

**Published:** 2024-04-18

**Authors:** Manon Van Hecke, Tom Langenaeken, Filip Rega, Tania Roskams, Bart Meuris

**Affiliations:** Department of Pathology, University Hospitals Leuven, Leuven, Belgium; Department of Cardiac Surgery, University Hospitals Leuven, Leuven, Belgium; Department of Cardiac Surgery, University Hospitals Leuven, Leuven, Belgium; Department of Pathology, University Hospitals Leuven, Leuven, Belgium; Department of Cardiac Surgery, University Hospitals Leuven, Leuven, Belgium

**Keywords:** Mechanical valves, Thrombogenicity, Large animal models, Preclinical, Valve design, Translational, Histopathology

## Abstract

**OBJECTIVES:**

Mechanical valves still require life-long anticoagulation. Preclinical animal testing is a crucial step in the assessment of valves; however, the chosen animal model should be carefully considered, and a well-controlled animal model of mechanical valve thrombosis has not been established yet. In this study, a histopathologic comparison was performed to evaluate the representativity of pigs and sheep as large animal models in bileaflet mechanical valve thrombosis research.

**METHODS:**

10 pigs and 8 sheep were implanted with a bileaflet mechanical valve in pulmonary position. During follow-up, no anticoagulative therapy was administered. Pigs were sacrificed between 14 and 38 days for explantation and assessment of the valve. Sheep were sacrificed between 71 and 155 days. Thrombus samples were processed and (immuno)histochemical stainings were applied. A pathologist evaluated the samples morphologically and semiquantitatively and compared these samples to available slides from 3 human patients who underwent redo surgery for acute bileaflet mechanical valve thrombosis, caused by insufficient anticoagulation.

**RESULTS:**

All pigs showed macroscopically evident thrombi on the mechanical valve surface at sacrifice. In contrast, none of the sheep showed any sign of thrombus formation. Histology showed a high fibrin content in thrombi of both human and porcine cases (3/3 vs 8/10). Porcine thrombi showed more cellular organization (0/3 vs 6/10), more calcification (0/3 vs 9/10) and more endothelialization (0/3 vs 6/10). Inflammatory cells were present in all samples and were considered physiological.

**CONCLUSIONS:**

Contrary to sheep, pigs develop thrombi on their mechanical valves in the short-term if no anticoagulation is administered. Histologic comparison of human and porcine thrombi shows comparable findings. The pig model might serve interestingly for further research on valve thrombosis, if it shows not to be an overly aggressive model.

## Graphical Abstract

**Figure:**
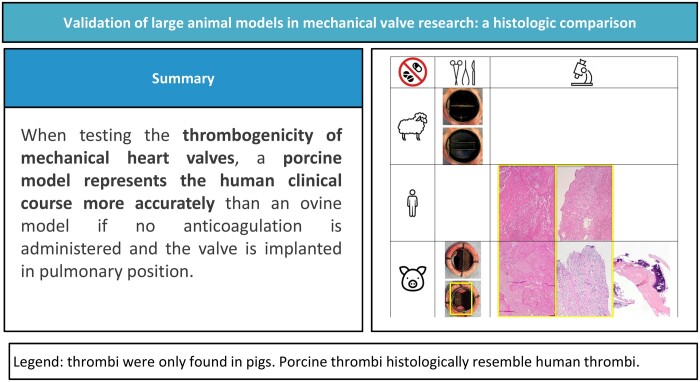

## INTRODUCTION

In the setting of terminal valve failure, mechanical prosthetic heart valves are still the most durable treatment option, given that proper anticoagulation is maintained. Currently, vitamin K antagonists are considered the only appropriate anticoagulative therapy, despite the need for careful international normalized ratio (INR) management and associated bleeding risk [1]. Alternatives such as direct oral anticoagulants failed in recent major trials [2, 3]. Animal models are essential in the preclinical study of heart valves, of which the most commonly used are sheep and pigs [4]. However, careful consideration of the haematological, anatomical and practical differences in animal species is of utmost importance. Sheep are considered the golden standard for the *in vivo* evaluation of implanted cardiovascular devices due to their comparable anatomy at adult age, wide availability at low price and ease of husbandry. However, the sheep as an adequate model for thrombogenicity testing has been questioned after the lessons learned from the failure of the Medtronic Parallel valve [5]. When specifically evaluating their coagulation system, differences with humans have been reported [6]. Pigs may provide a valid alternative, despite their rapid growth making them less suited for prolonged follow-up studies [7]. *In vitro* assessment of the porcine platelet response on mechanical heart valve leaflets showed more similarities with the human platelet response than the ovine platelet response did [8, 9].

This study evaluated the *in vivo* relevance of sheep and pigs as large animal models to study bileaflet mechanical valve thrombosis by reporting macroscopic findings at necropsy and by providing a detailed histologic inter-species comparison.

## MATERIALS AND METHODS

ARRIVE guidelines were applied for the reporting of this study [10].

### Ethical statement

The local Ethics Committee approved the study (Ethische Commissie Dierproeven, KU Leuven, Study number S67560, on 26 April 2023) and waived the obtaining of patients’ written informed consent.

### Experiment design

A total of 10 male neutered Landrace × Yorkshire pigs and 8 female adult Swifter sheep were acquired from the ZOOtechnical center (ZTC, Lovenjoel, Belgium) and cared for at the animal care facility of the University of Leuven under supervision of a veterinarian, in accordance with the Guide for the Care and Use of Laboratory Animals, 8th Edition, formulated by the National Research Council [11]. All animals were housed for a minimum of 1 week before valve implantation. No sample size calculation was performed for this study. The follow-up period was 20 weeks, or until valve failure due to thrombosis or other reasons. All valves used were 21-mm bileaflet mechanical valves. In the pig cohort, 6 animals received St Jude Medical Mechanical Heart Valve (St Jude Medical Inc.; Minneapolis, MN), 2 On-X mechanical heart valve (CryoLife and BicarbonVR, LivaNova) and 2 Carbomedics Top Hat (Sulzer, Carbomedics, Austin, TX). In the sheep cohort, 2 animals received the St Jude Medical Mechanical Heart Valve (St Jude Medical Inc.; Minneapolis, MN) and 6 animals received the On-X (On-X, CryoLife and BicarbonVR, LivaNova). All valves were implanted in pulmonary position.

### Valve implantation

All animals were fasted 24 h prior to surgery. Sheep were sedated using ketamine by jugular venous injection. Pigs were sedated using intramuscular midazolam and xylazine. An arterial pressure line was inserted in the right ear and a venous access line was inserted in left upper leg. Anaesthesia was induced and maintained by intravenous propofol. Positive pressure ventilation was started after intubation. A large bore gastric tube for gastric decompression was placed. Antibiotic prophylaxis by ceftiofur was given according to animal weight. Animals were positioned in right lateral recumbent position followed by surgical scrubbing and draping to expose the left cervical region and left chest wall.

A left anterolateral thoracotomy was made in the 3rd intercostal space. Lidocaine was given for arrhythmia prevention. The pericardium was incised and cradled by single sutures. Care was made to spare the phrenic nerve. In the sheep, normothermic cardiopulmonary bypass was established by canulation of the left neck vessels (carotid artery and jugular vein) after heparinization (heparin sodium, 250 IU/kg bodyweight; Rhone-Poulenc Rorer, Brussels, Belgium). A target activating clotting time of 350 s was reached and maintained. In the pigs, normothermic cardiopulmonary bypass was established by cannulating the ascending aorta and right atrial appendage after dissection of the aortopulmonary window and heparinization (300 IU/kg, target activating clotting time >500).

The distal pulmonary artery was clamped once sufficient venous drainage was achieved. The pulmonary trunk was opened at the level of the sinotubular junction. Supplementary venous drainage was achieved by inserting a sump catheter in the right ventricle trough the pulmonary valve ensuring a dry working field. The native valve leaflets were excised and the prosthetic heart valve was implanted orthotopically in pulmonary position. The pulmonary trunk was closed with a single running suture after proper de-airing and checking the valve position and function. The animals were decannulated once adequate weaning of cardiopulmonary bypass was achieved and haemodynamic parameters were stable. The pericardium was left open and the chest closed in standard fashion with resorbable sutures. One pleural drain was placed. The jugular incision was closed in layers with resorbable sutures.

### Postoperative care and echocardiography

The chest drain was removed ∼1–2 h after surgery, when sedation was fading and the animal was breathing adequately. Feeding was allowed immediately. Adequate analgesia (sheep: piritramide, pigs: meloxicam and buprenorphine), diuretics (pigs and sheep: furosemide) and antibiotics (sheep: ampicillin, pigs: ceftiofur) were given during the first 2 postoperative days. During the 1st postoperative week, low-molecular-weight heparin (pigs and sheep: enoxaparin 20 mg twice daily) was administered by subcutaneous injection. At the end of the 1st week, all medication and anticoagulation was stopped.

All animals underwent monthly transthoracic echocardiography to evaluate visual opening and closure of the cusps, transvalvular gradients and regurgitation. If a clear rise in gradients and/or a clear increase in valvular regurgitation was seen, X-ray fluoroscopy was used to confirm standstill of 1 or both cusps.

### Explant procedures

At valve thrombosis, identified by total absence of any valve movement on cardiac ultrasound and/or X-ray fluoroscopy, or at follow-up completion, animals were euthanized with pentobarbital after heparinization (heparin 300 IU/kg) to prevent post-mortem thrombosis. A cardiectomy was performed. The valves were carefully dissected form the surrounding tissue and assessed for evidence of structural degradation, thrombus, vegetation, pannus overgrowth, hinge integrity and paravalvular leak. If tissue was present, it was removed from the valve surface, weighed and sent for histologic assessment. The lungs, as the 1st downstream capillary bed, were macroscopically evaluated and random lung biopsies were taken to screen for thrombo-embolic damage.

### Human control samples

Three human cases were carefully selected for comparison of the obtained thrombus tissue. Only patients with implantation of a bileaflet mechanical heart valve and without history of endocarditis were considered. The selected human patients underwent redo surgery for acute valve thrombosis, caused by insufficient anticoagulation upon clinical assessment.

In the 1st case, the patient was treated with a 31-mm St Jude Medical Mechanical Heart Valve (St Jude Medical Inc.; Minneapolis, MN) implanted in tricuspid position in 1994 and went home with a Vitamin K antagonist-based anticoagulation scheme. In 2004, this patient visited the emergency department because of dyspnoea and absent prosthetic valve noises that started 1 day before his visit. No details were noted regarding the patients’ medication intake. Analysis at the emergency department revealed an INR of 1.5.

The 2nd selected patient received a 33-mm On-X mechanical heart valve (CryoLife and BicarbonVR, LivaNova) in mitral position in 2018 and went home with a Vitamin K-based anticoagulation scheme. Eighteen months later, this patient visited the emergency department because of dyspnoea since 1 week. No details were noted regarding the patients’ medication intake. Assessment at the emergency department revealed an INR of 1.4 and peripheral and pulmonary embolism.

The final patient was treated with a 27-mm Carbomedics Top Hat (Sulzer, Carbomedics, Austin, TX) in aortic position in 2019 and went home with a Vitamin K-based anticoagulation scheme. In 2022, this patient visited the emergency department with symptoms of dyspnoea, palpitations and chest pain since several weeks. An INR value was not reported, nor were there details regarding the patients’ medication intake, since the reports are incomplete because the patient left the hospital on their own initiative.

### Histology

All porcine thrombus samples were collected and stored in formalin for at least 24 h, and subsequently embedded in paraffin. Of each tissue block, five 5-µm slides were cut and stained with haematoxylin–eosin (Leica Biosystems, Wetzlar, Germany), Picrosirius Red (without counterstain; Polysciences, Hirschberg an der Bergstraße, Germany and VWR, Radnor, PA, USA) and with commercially available antibodies against CD45 (Abcam, Cambridge, UK) CD31 (Abcam, Cambridge, UK) and alpha-smooth muscle actin (aSMA) (Dako, Glostrup, Denmark), after optimizing each staining protocol for porcine tissue in our lab. All collected lung samples were stored in formalin for at least 24 h, and subsequently embedded in paraffin. Of each tissue block, five 5-µm slides were cut and stained with haematoxylin–eosin (Leica Biosystems, Wetzlar, Germany).

The human thrombi samples, obtained during redo surgery, had been formalin-fixed and paraffin-embedded for routine diagnostics in the pathology department of UH Leuven in the past. Therefore, the tissue block and haematoxylin–eosin slide were available from the UH Leuven archives. Four additional 5-µm slides were cut from the formalin-fixed and paraffin-embedded block and stained with Picrosirius Red (without counterstain; Polysciences, Hirschberg an der Bergstraße, Germany and VWR, Radnor, PA, USA), anti-CD45 (Dako, Glostrup, Denmark), anti-CD31 (Dako, Glostrup, Denmark) and anti-aSMA (Dako, Glostrup, Denmark) using standardized immunohistochemistry protocols.

All obtained slides from porcine and human thrombi were morphologically examined and blindly scored by a dedicated, experienced pathologist using light microscopy. The scoring system used for this study (Table 1) is an adaptation from a scoring system developed by CVPath (Gaithersburg, MD, USA), and represents the composition of the scored thrombus based on its fibrin content, the formation of pannus, collagen or calcifications, the presence of inflammatory cells and the extent of endothelialization, hereby estimating its degree of organization and thus its age. This approach allows for a both qualitative and a semi-quantitative comparison between porcine and human thrombi.

**Table 1:** Scoring system for semi-quantitative assessment of each thrombus, based on a scoring system designed by CVPath (Gaithersburg, Maryland, USA) and applied blindly by a dedicated pathologist

Attribute	Score	Description of assigned score
Unorganized fibrin content	0	No fibrin
	1	Minimal fibrin involving <10% of the material
	2	Mild fibrin involving 10–25% of the material
	3	Moderate fibrin involving 25–50% of the material
	4	Severe fibrin involving >50% of the material
Pannus formation	0	No pannus formation
	1	Minimal pannus involving <10% of the material
	2	Mild pannus involving 10–25% of the material
	3	Moderate pannus involving 25–50% of the material
	4	Severe pannus involving 50–75% of the material
	5	Heavy pannus involving >75% of the material
Degree of calcification	0	No calcification
	1	Minimal calcification involving <1% of the material
	2	Mild calcification involving 1–5% of the material
	3	Moderate calcification involving 5–10% of the material
	4	Severe calcification involving >10% of the material
Presence of inflammatory cells	0	Absent
	1	Mild
	2	Marked
Endothelialization	0	No endothelium
	1	Endothelium present

## RESULTS

### Perioperative outcome and survival

All animals remained healthy throughout the study period and survived. Regular veterinary inspections did not report complications. All data were therefore included in the study.

### Necropsy

**Figure 1:**
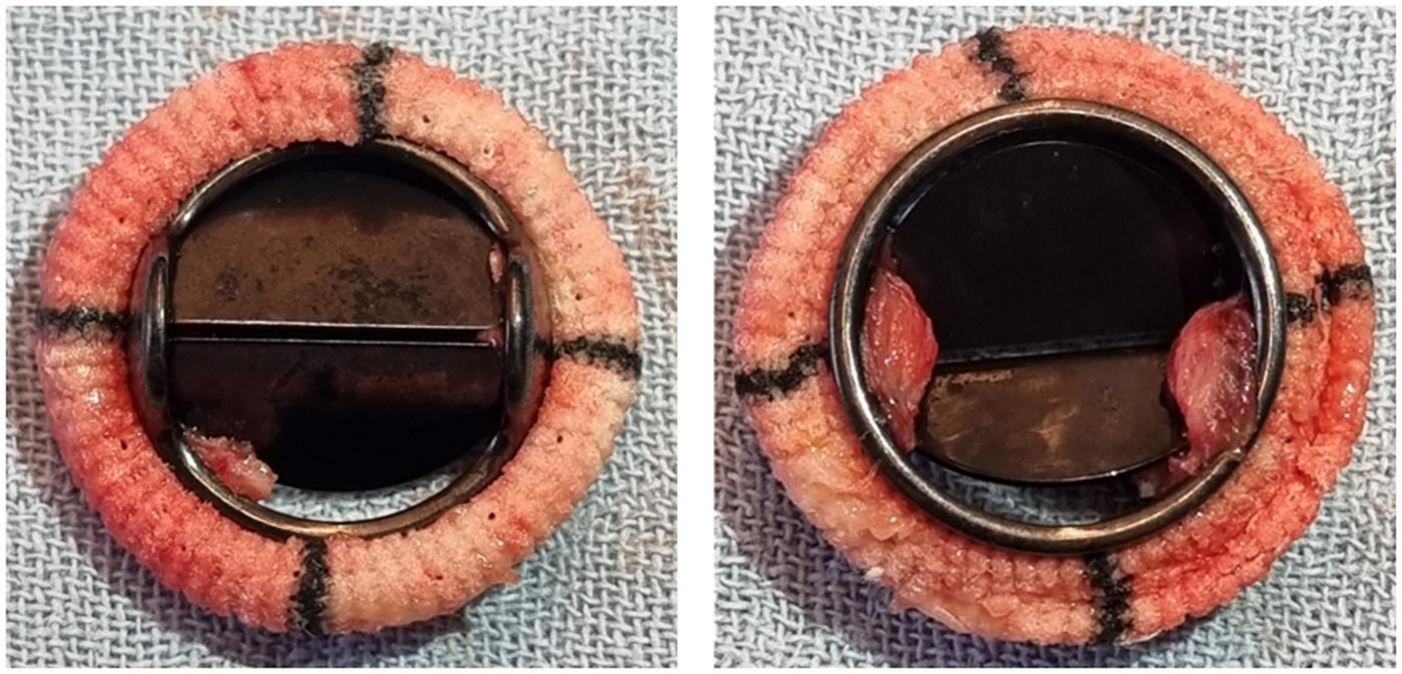
Macroscopic aspect of a representative thrombus formed on the bileaflet mechanical valve in one of the pigs with pulmonary (left panel) and ventricular (right panel) side.

In all 10 pigs, valve thrombosis occurred between 14 and 38 days of implantation. Necropsy revealed formation of macroscopically obvious thrombi on the mechanical valve surface, primarily at the hinges. The thrombus fixed the valve cusps in a partially open position, requiring forceps manipulation for mobilization of the cusps. All thrombi had a pink appearance (Fig. 1). The weight of the individual thrombi varied between 0.0121 and 0.408 g.

**Figure 2:**
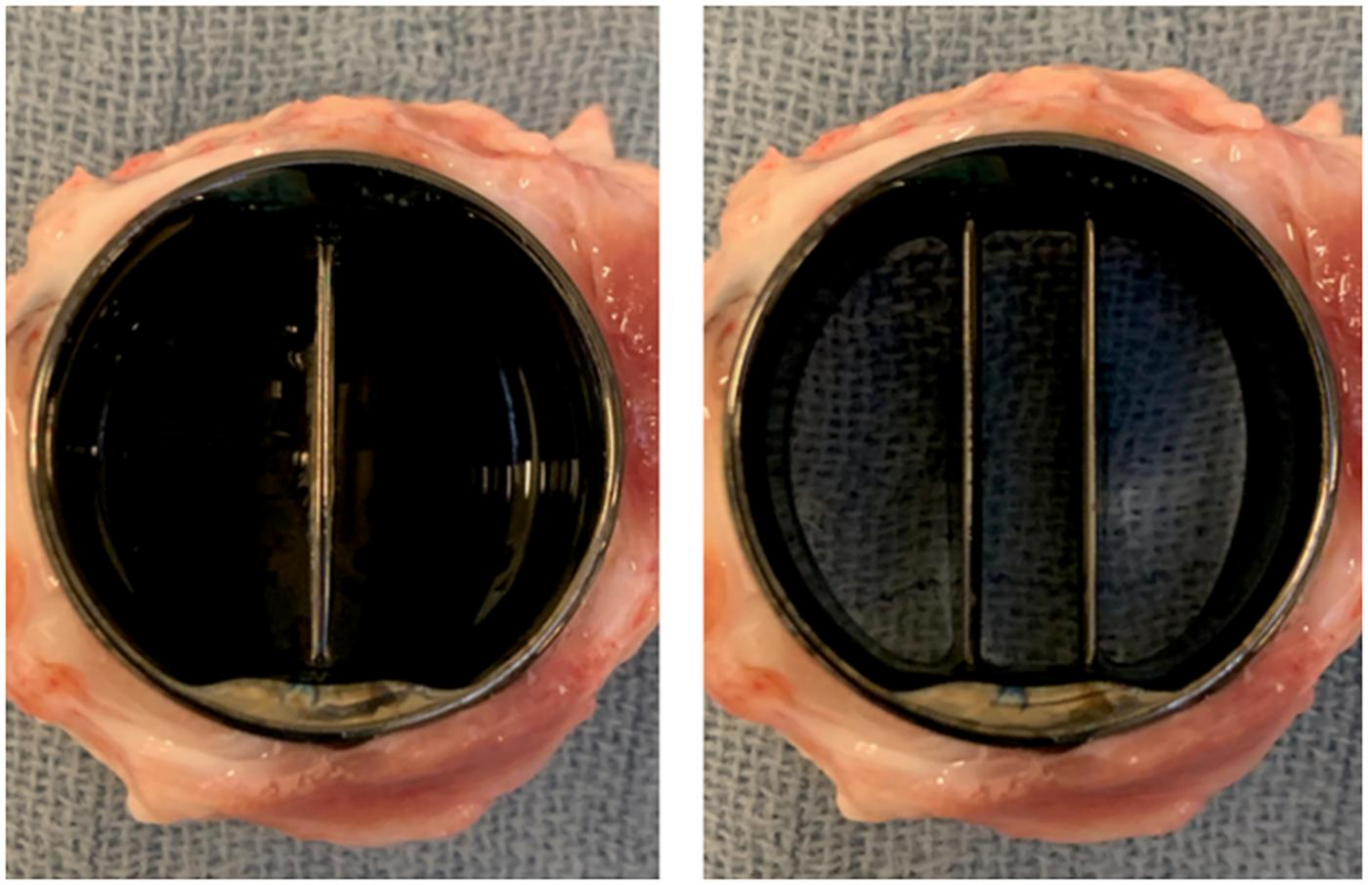
Macroscopic aspect of a representative, clean bileaflet mechanical valve in one of the sheep with pulmonary (left panel) and ventricular (right panel) side.

In contrast, all 8 sheep completed the follow-up period without any signs of thrombosis on the mechanical valve surface, with mobile cusps opening and closing without any resistance (Fig. 2).

### Histology

**Figure 3:**
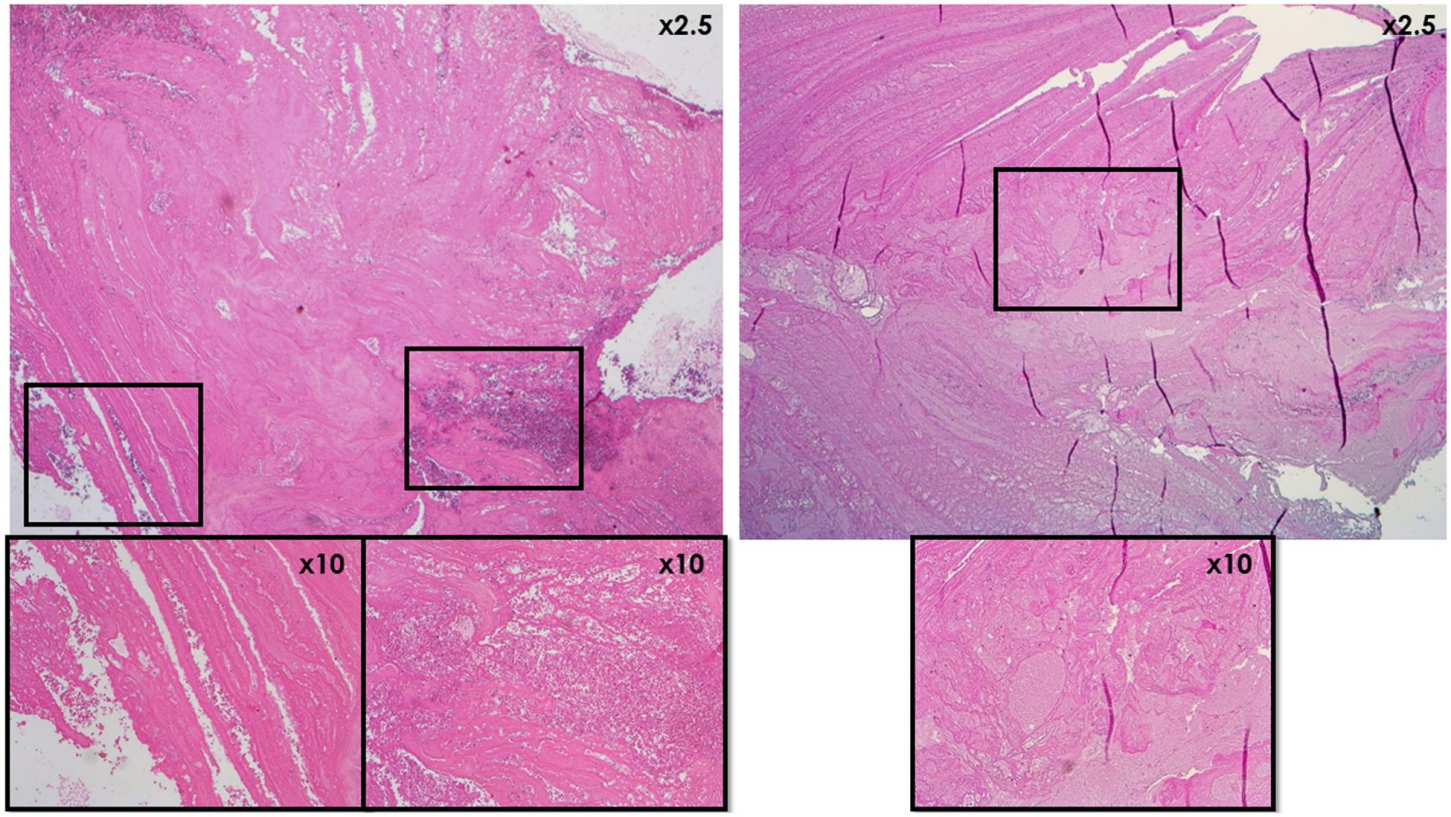
Microscopic haematoxylin–eosin-stained comparison of the fibrin content in human (left 3 panels) and porcine (right 2 panels) thrombi. The overview images (×25 magnification) clearly show the layered appearance in both cases. The detailed images (×100 magnification) show deposition of intensely eosinophilic fibrin associated with platelets, with focal presence of red blood cells in the human thrombus. The porcine thrombus consists of fibrin and platelets only, while the red blood cells have been degraded.

**Figure 4:**
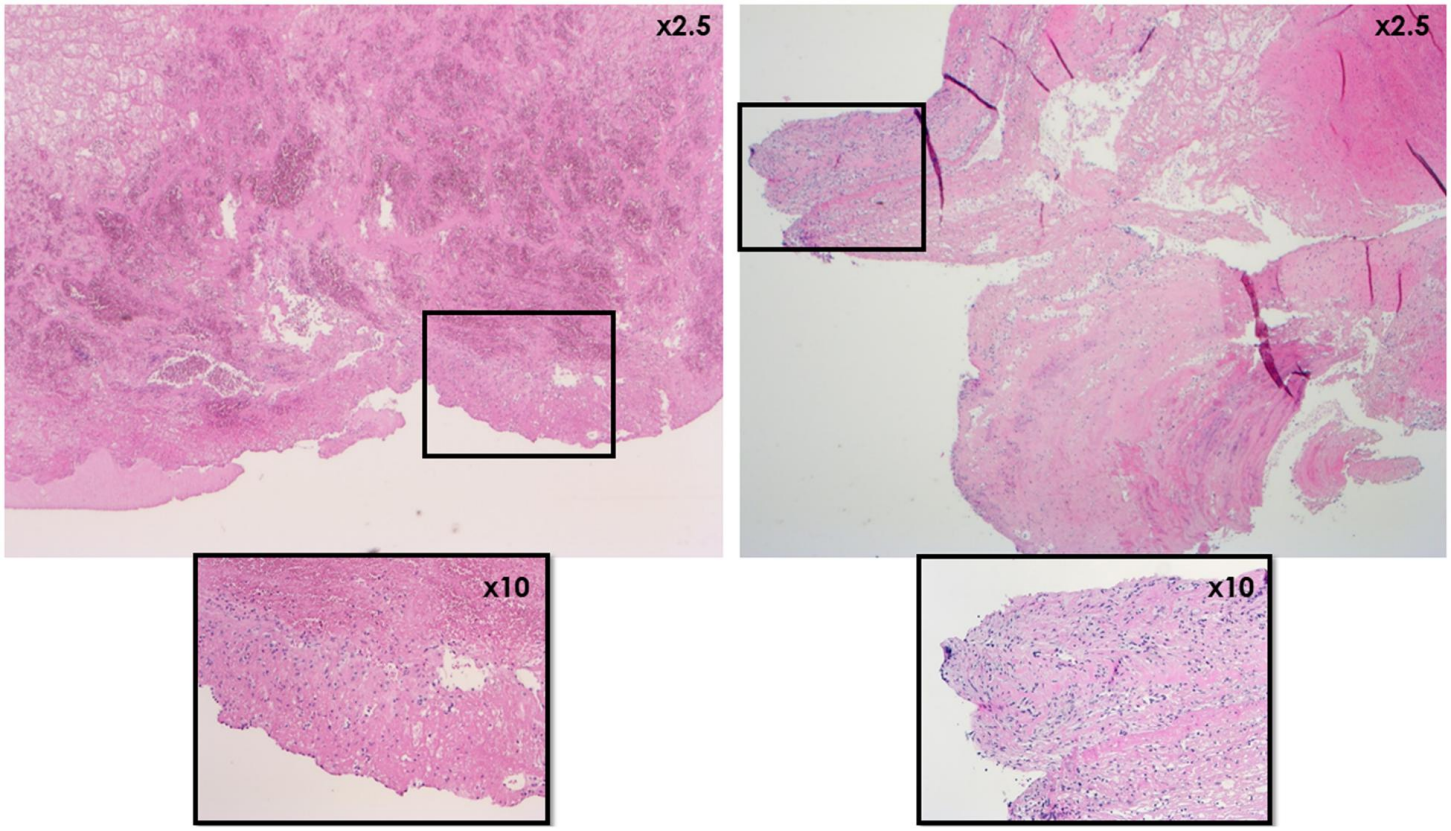
Microscopic haematoxylin–eosin-stained comparison of pannus formation in human (left 2 panels) and porcine (right 2 panels) thrombi. The overview images (×25 magnification) show focal cellular organization at the edges of the thrombi. The detailed images (×100 magnification) confirm the infiltration of spindled cells in these foci.

**Figure 5:**
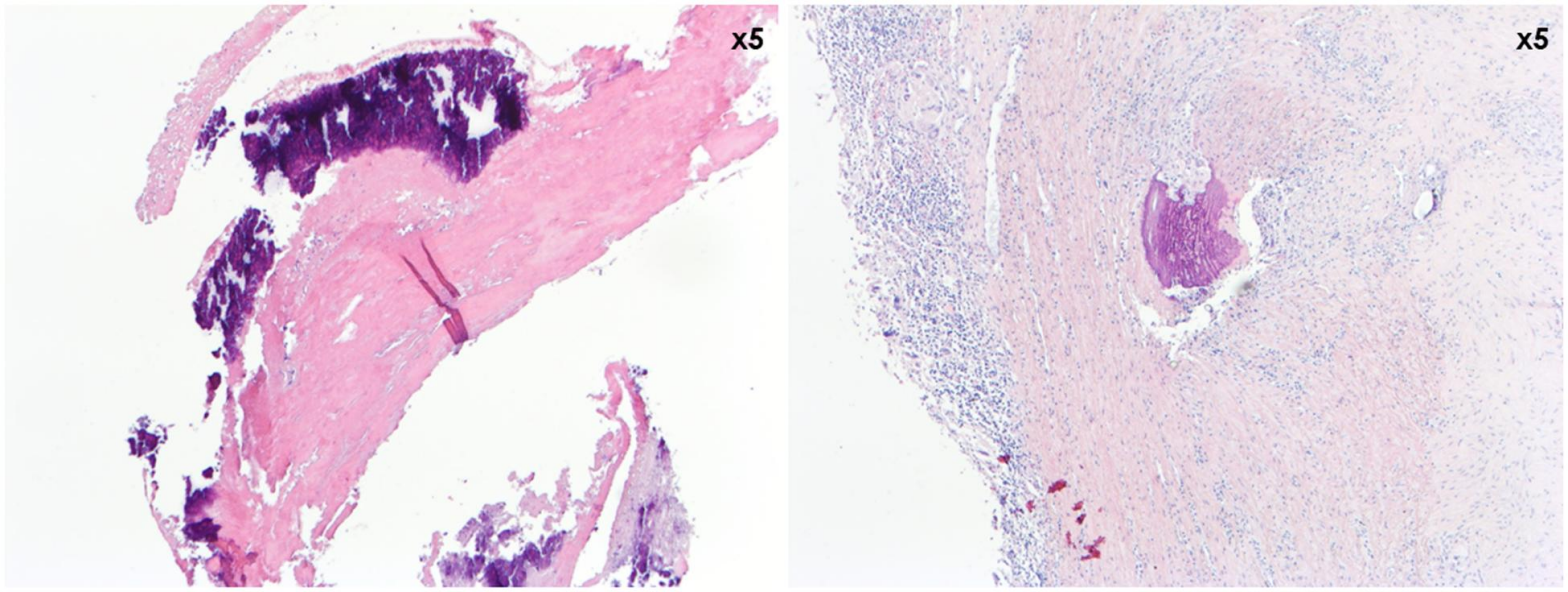
Overview haematoxylin–eosin-stained images (×50 magnification) of intense purple staining calcifications found in the porcine thrombi. Note that calcification can occur in the unorganized fibrin stage (left panel) as well as in the stage of cellular organization and remodelling (right panel) due to dystrophia.

None of the investigated lung samples showed signs of thrombo-embolism. Since no thrombi were found in sheep, only the porcine (*n* = 10) and human (*n* = 3) thrombi were investigated microscopically. None of the investigated thrombi showed signs of infection. Thrombi of pigs and humans were mostly composed of layers of unorganized fibrin and platelets (Fig. 3), in the human cases interspersed with layers of fresh red blood cells (‘lines of Zahn’). The semi-quantitative scoring (Table 2) confirmed a fibrin content of >50% in 3/3 human and 8/10 porcine samples, with the remaining 2 porcine samples containing 25–50% or 10–25% fibrin content. The human thrombi did not show obvious ingrowth of cellular elements yet except for some very limited foci, whereas partial pannus formation was observed in multiple porcine thrombi (Fig. 4). Blinded assessment verified the lack of pannus formation in 3/3 human and 4/10 porcine thrombi. In the pig cohort, 4/10 porcine thrombi showed limited cellular organization of <10% of the sample, 1/10 showed 50–75% and 1/10 showed >75% pannus formation. Pannus formation (in the pig cohort) was further confirmed by the presence of aSMA-positive cells, depositing collagen staining with Picrosirius Red. There were no calcifications observed in the human samples, whereas the porcine thrombi showed calcified foci (Fig. 5). Indeed, 0/3 human cases revealed calcification upon scoring. In contrast, 3/10 pigs scored <1% calcification, 2/10 scored 1–5%, 1/10 scored 5–10% and 3/10 scored >10% calcification of the total sample. The presence of white blood cells is apparent in all human and porcine samples, either randomly entrapped in fibrin or actively functioning in the remodelling reaction at the pannus, in mild (2/3 human and 7/10 porcine samples) or marked (1/3 human and 3/10 porcine samples) quantities. Note that this is a physiologic finding in this context, not associated with infection. Finally, endothelialization of parts of the thrombi was observed in 6/10 pigs but not in the human samples.

**Table 2:** Results of the blinded scoring of each thrombus

Case	Species	Unorganized fibrin content	Pannus formation	Degree of calcification	Presence of inflammatory cells	Endothelialization
1	Human	4	0	0	2	0
	Pig	4	1	1	1	1
2	Human	4	0	0	2	0
	Pig	4	0	4	2	1
3	Human	4	0	0	1	0
	Pig	4	0	1	1	0
4	Pig	4	1	2	1	0
5	Pig	4	1	4	1	1
6	Pig	4	0	4	2	0
7	Pig	4	1	3	2	1
8	Pig	4	0	0	1	0
9	Pig	3	4	2	1	1
10	Pig	2	5	1	1	1

## DISCUSSION

The findings of this study are two fold: first, macroscopic assessment at necropsy revealed that pigs develop thrombi on bileaflet mechanical valves, implanted in pulmonary position, on the short-term if no anticoagulation is administered, while sheep do not. Second, the thrombi formed in pigs are histologically of comparable composition as human thrombi, with porcine thrombi showing a more advanced stage of organization.

It is assumed that valves implanted in the right heart are more prone to develop thrombosis because of different haemodynamics compared to the left side [12]. The lower washout of the hinges of the mechanical valve due to lower pressure gradients in the right-sided heart undoubtedly contributes to a more thrombogenic environment. Furthermore, there a difference in prostacyclin levels in arterial and venous blood, which is a powerful inhibitor of platelet aggregation [13]. With prostacyclin less present in venous blood, this may be an explanation. The decision to implant the prosthetic valves in pulmonary position in our animals was also based on previous work, which showed that it is a reliable and reproducible model to study mechanical valve thrombosis [14, 15]. Mechanical valves orthotopically implanted on the left side in either aortic or mitral position do not easily show valve thrombosis, but it would be interesting to test mitral and aortic valve replacement in future porcine experiments for optimization of this preclinical model. Additionally, in our experience, left-sided valve interventions in pigs are associated with higher mortality and morbidity, so these challenges need to be addressed first.

The observation that thrombosis occurs repetitively in our pigs but not in our sheep is mainly attributable to inter-species differences in haematology. From the moment that a mechanical heart valve, or any other material, is implanted in contact with the bloodstream, adsorption of plasma proteins to the synthetic surface occurs, altering the shape of the protein molecule itself. The circulating platelets have a certain affinity for these altered, adsorbed proteins through their receptors, enabling platelet activation, followed by platelet aggregation and activation of the blood coagulation cascade along with complement and inflammatory factors [16]. Inter-species differences in the coagulation system have been studied only in a few experiments, with ovine platelets exhibiting less attachment, aggregation, spread and subsequent thrombus growth on the biomaterial surface in comparison to human or porcine platelets [8, 17, 18]. Aside from the platelet response, there is evidence for direct adsorption of factor XII to synthetic surfaces in contact with the blood stream with subsequent autoactivation of the intrinsic blood coagulation cascade, with the 2 mechanisms interacting synergistically [16].

**Figure 6:**
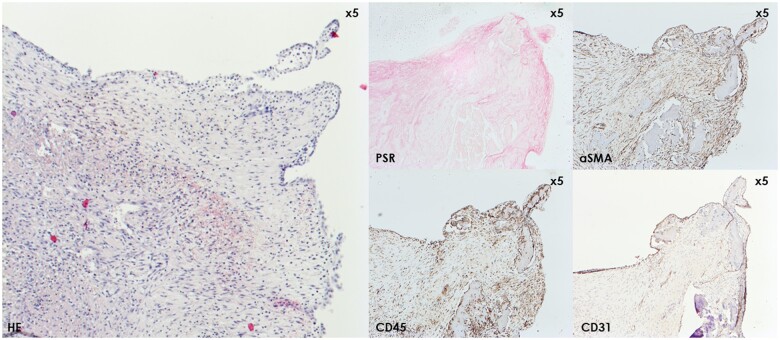
Illustration (×50 magnification) of immunohistochemical findings in porcine pannus with haematoxylin–eosin stain (left panel) for reference. Pannus is marked by population of the thrombus by myofibroblasts (aSMA-positive) that will actively form collagen, as confirmed by the red threads shown by the PSR stain. The CD45-positive inflammatory cells work together with the myofibroblasts to remodel the thrombus into stable connective tissue with newly formed blood vessels. The surface of this connective tissue gets covered by a single layer of endothelial cells, as can be seen in the CD31 stain. The end product is a neointima. aSMA: alpha-smooth muscle actin; PSR: Picrosirius Red.

The end product of this cascade is a thrombus composed of a meshwork of fibrin, platelets and red blood cells, which we could confirm microscopically in all our human and porcine samples, and which has been reported previously [19–22]. From this point, the thrombus can theoretically either propagate, embolize, dissolve or organize over time; 4 events that in practice mostly happen simultaneously if the patient survives and if no intervention takes place. The process of thrombus organization deserves specific attention. This process is characterized by the ingrowth of cellular elements from the nearby healthy vessel wall; the fibrin clot gradually gets infiltrated by spindled (myo)fibroblasts and macrophages, working together to remodel the clot into a stable, collagenous neointima, which will ultimately get covered by endothelium (Fig. 6). Calcifications can develop at any time during this remodelling due to the dystrophic environment [23]. In our human samples, only limited foci of early organization were visible, while it was more frequently and more extensively observed in the porcine samples. Although it is likely that this difference too is attributable to species differences, it is also a time-dependent process. Indeed, 2 of our human patients underwent urgent redo surgery upon symptomatic presentation, while the earliest sacrifice of the pigs was only after 14 days post-implantation. Altogether, the microscopic assessment confirms that the macroscopically observed thrombi of humans and pigs are of comparable composition, and that the same pathophysiologic process seems to occur in both species in this context.

Moreover, the description of neointima given before, also perfectly matches the histologic features of pannus tissue. Clinically, thrombus and pannus are often considered 2 distinct entities, both precipitating prosthetic valve obstruction. This distinction is of relevance since fresh thrombi can sometimes be managed by fibrinolysis alone, while pannus nearly always requires surgical replacement. But, organized thrombus and pannus could very well reflect a later stage of the same tissue reaction, which has been suggested before; not only does their histologic appearance overlap, both occur through the same haemodynamic and haemostatic triggers, and are not unfrequently found simultaneously [24–30].

It should be noted that the pig model still comes with its own challenges. Due to rapid growth of these animals, size mismatch of the implanted valve can cause elevated gradients and turbulent flow. In our study, we did not experience this, since all pigs were all euthanized within 38 days, which is probably too short for size mismatches to occur. Furthermore, the coagulation system of pigs has been reported to be hypercoagulable in comparison to humans, and proper anticoagulation is difficult to obtain in pigs [6].

### Limitations

Although it would have been ideal if one and the same valve type had been used all cases, the type of bileaflet mechanical valve implanted in the animals was mainly chosen by its availability combined with its clinical relevance. Indeed, when selecting suitable human cases, these chosen types of bileaflet valves were represented. Another difference to be aware of is the fact that the human patients were treated for an unknown period of time with vitamin K-antagonists prior to their redo surgery. In contrast, our animals received low-molecular-weight heparins for 1 week only, after which all anticoagulation was stopped for the remaining follow-up period. Therefore, the environment in which the thrombi formed might be different. Nevertheless, microscopic assessment of porcine and human thrombi revealed interesting similarities.

## CONCLUSION

Without anticoagulative therapy, pigs develop thrombi on bileaflet mechanical valves implanted in the pulmonary position in the short-term, whereas sheep do not (not even on the long term). Since all valves thrombosed quite rapidly, the pig model might be an overly aggressive model, which has to be evaluated in further studies. Histologic examination of human and porcine thrombi shows comparable pathophysiologic findings. Hence, for the assessment of thrombogenicity of mechanical heart valves, and even other implanted cardiovascular devices, pigs may be the better animal model compared to sheep.

## Data Availability

All data are incorporated into the article.

## Notes

Presented at the 37th EACTS Annual Meeting, Vienna, Austria, 4–7 October 2023.

## ABBREVIATION

INR: International normalized ratio

## References

[1] Head SJ , Çelik M, Kappetein AP. Mechanical versus bioprosthetic aortic valve replacement. Eur Heart J 2017;38:2183–91. doi: 10.1093/eurheartj/ehx141. 28444168

[2] Eikelboom JW , Connolly SJ, Brueckmann M, Granger CB, Kappetein AP, Mack MJ et al; RE-ALIGN Investigators. Dabigatran versus warfarin in patients with mechanical heart valves. N Engl J Med 2013;369:1206–14. doi: 10.1056/NEJMoa1300615. Epub 2013 31. 23991661

[3] Wang TY , Svensson LG, Wen J, Vekstein A, Gerdisch M, Vijay UR et al Apixaban or warfarin in patients with an On-X mechanical aortic valve. NEJM Evid 2023;2.EVIDoa2300067. doi: 10.1056/EVIDoa2300067. 38320162

[4] Gallegos RP , Nockel PJ, Rivard AL, Bianco RW. The current state of *in vivo* pre-clinical animal models for heart valve evaluation. J Heart Valve Dis 2005;14:423–32. 15974538

[5] Bodnar E. The medtronic parallel valve and the lessons learned. J Heart Valve Dis 1996;5:572–3. 8953433

[6] Mizuno T , Tsukiya T, Takewa Y, Tatsumi E. Differences in clotting parameters between species for preclinical large animal studies of cardiovascular devices. J Artif Organs 2017;21:138–41. doi: 10.1007/s10047-017-1003-4. Epub 2017 9. 29124459

[7] Byrom MJ , Bannon PG, White GH, Ng MK. Animal models for the assessment of novel vascular conduits. J Vasc Surg 2010;52:176–95. doi: 10.1016/j.jvs.2009.10.080. Epub 2010 29. 20299181

[8] Goodman SL. Sheep, pig, and human platelet-material interactions with model cardiovascular biomaterials. J Biomed Mater Res 1999;45:240–50. doi: 10.1002/(sici)1097-4636(19990605)45:3<240::aid-jbm12>3.0.co;2-c. 10397982

[9] Goodman SL , Tweden KS, Albrecht RM. Platelet interaction with pyrolytic carbon heart-valve leaflets. J Biomed Mater Res 1996;32:249–58. doi: 10.1002/(SICI)1097-4636(199610)32:2<249::AID-JBM15>3.0.CO;2-E. 8884503

[10] Percie Du Sert N , Hurst V, Ahluwalia A, Alam S, Avey MT, Baker M et al The ARRIVE Guidelines 2.0: updated guidelines for reporting animal research. PLoS Biol 2020;18.e3000410. doi: 10.1371/journal.pbio.3000410. 32663219 PMC7360023

[11] National Research Council (US) Committee For the Update of the Guide for the Care and Use of Laboratory Animals. Guide for the Care and Use of Laboratory Animals. 8th edn. Washington, DC: National Academies Press (US), 2011. doi: 10.17226/12910. 21595115

[12] Roudaut R , Serri K, Lafitte S. Thrombosis of prosthetic heart valves: diagnosis and therapeutic considerations. Heart 2007;93:137–42. doi: 10.1136/hrt.2005.071183. 17170355 PMC1861363

[13] Péterffy Á , Szentkirályi I. Mechanical valves in tricuspid position: cause of thrombosis and prevention. Eur J Cardiothorac Surg 2001;19:735–6. doi: 10.1016/S1010-7940(01)00667-4 11432368

[14] Meuris B. Research on biological and mechanical heart valves: experimental studies in chronic animal models. Verh K Acad Geneeskd Belg 2002;64:287–302. 12416236

[15] Meuris B , Verbeken E, Flameng W. Mechanical valve thrombosis in a chronic animal model: differences between monoleaflet and bileaflet valves. J Heart Valve Dis 2005;14:96–104. 15700443

[16] Wagner WR , Sakiyama-Elbert SE, Zhang G, Yaszemski MJ. Biomaterials science : an introduction to materials in medicine. Philadelphia, PA: Elsevier, 2020.

[17] Tillman P , Carson SN, Talken L. Platelet function and coagulation parameters in sheep during experimental vascular surgery. Lab Anim Sci 1981;31:263–7. 7265903

[18] Weigand A , Boos AM, Ringwald J, Mieth M, Kneser U, Arkudas A et al New aspects on efficient anticoagulation and antiplatelet strategies in sheep. BMC Vet Res 2013;9:192. doi: 10.1186/1746-6148-9-192. 24088206 PMC3851128

[19] Chitturi KR , Castro MA, Salazar E, Deavers M, Chang SM, Mahmarian JJ et al Mechanical mitral valve thrombosis in a patient with prior nonbacterial thrombotic endocarditis. JACC Case Rep 2020;2:539–43. doi: 10.1016/j.jaccas.2020.01.029. 34317289 PMC8298676

[20] Kreuziger LB , Slaughter MS, Sundareswaran K, Mast AE. Clinical relevance of histopathologic analysis of HeartMate II thrombi. Asaio J 2018;64:754–9. doi: 10.1097/MAT.0000000000000759. 29461277 PMC6093800

[21] Scheidmann R , Foth R, Sigler M. Thrombosis of a mechanical prosthetic aortic valve in early pregnancy: histopathological findings. Cardiovasc Pathol 2017;27:35–6. doi: 10.1016/j.carpath.2016.12.003 28081513

[22] Ávila WS , Caldas VAFC, Batista DV, Gutierrez PS. Case 5/2018 – Acute respiratory failure and cardiogenic shock in a patient in the first trimester of pregnancy with mechanical mitral valve prosthesis implant. Arq Bras Cardiol 2018;111:629–34. doi: 10.5935/abc.20180205. 30365686 PMC6199511

[23] Kumar V , Abbas AK, Aster JC. Robbins Basic Pathology, 10th edn. Philadelphia, PA: Elsevier, 2021:115–40.

[24] Dangas GD , Weitz JI, Giustino G, Makkar R, Mehran R. Prosthetic heart valve thrombosis. J Am Coll Cardiol 2016;68:2670–89. doi: 10.1016/j.jacc.2016.09.958. 27978952

[25] Salamon J , Munoz-Mendoza J, Liebelt JJ, Taub CC. Mechanical valve obstruction: review of diagnostic and treatment strategies. World J Cardiol 2015;7:875–81. doi: 10.4330/wjc.v7.i12.875. 26730292 PMC4691813

[26] Barbetseas J , Nagueh SF, Pitsavos C, Toutouzas PK, Quiñones MA, Zoghbi WA. Differentiating thrombus from pannus formation in obstructed mechanical prosthetic valves: an evaluation of clinical, transthoracic and transesophageal echocardiographic parameters. J Am Coll Cardiol 1998;32:1410–7. doi: 10.1016/s0735-1097(98)00385-4. 9809956

[27] Vitale N , Renzulli A, Agozzino L, Pollice A, Tedesco N, de Luca Tupputi Schinosa L et al Obstruction of mechanical mitral prostheses: analysis of pathologic findings. Ann Thorac Surg 1997;63:1101–6. doi: 10.1016/s0003-4975(96)01391-4. 9124913

[28] Aoyagi S , Nishimi M, Tayama E, Fukunaga S, Hayashida N, Akashi H et al Obstruction of St Jude medical valves in the aortic position: a consideration for pathogenic mechanism of prosthetic valve obstruction. Cardiovasc Surg 2002;10:339–44. doi: 10.1016/s0967-2109(02)00021-2. 12359404

[29] Renzulli A , De Luca L, Caruso A, Verde R, Galzerano D, Cotrufo M. Acute thrombosis of prosthetic valves: a multivariate analysis of the risk factors for a lifethreatening event. Eur J Cardiothorac Surg 1992;6:412–20; discussion 421. doi: 10.1016/1010-7940(92)90065-6. 1389247

[30] Teshima H , Hayashida N, Yano H, Nishimi M, Tayama E, Fukunaga S et al Obstruction of St Jude Medical valves in the aortic position: histology and immunohistochemistry of pannus. J Thorac Cardiovasc Surg 2003;126:401–7. doi: 10.1016/s0022-5223(03)00702-5. 12928636

